# Artificial olfactory sensor technology that mimics the olfactory mechanism: a comprehensive review

**DOI:** 10.1186/s40824-022-00287-1

**Published:** 2022-08-19

**Authors:** Chuntae Kim, Kyung Kwan Lee, Moon Sung Kang, Dong-Myeong Shin, Jin-Woo Oh, Chang-Soo Lee, Dong-Wook Han

**Affiliations:** 1 Bionanotechnology Research Center, Korea Research Institute of Bioscience and Biotechnology (KRIBB), Daejeon, 34141 South Korea; 2 Department of Biomedical and Nanopharmaceutical Sciences, Graduate School, Kyung Hee University, Seoul, 02447 South Korea; 3 Department of Cogno-Mechatronics Engineering, Pusan National University, Busan, 46241 South Korea; 4 Department of Mechanical Engineering, The University of Hong Kong, Pokfulam, 999077 Hong Kong, China; 5 Department of Nanoenergy Engineering, Pusan National University, Busan, 46241 South Korea; 6 Department of Biotechnology, University of Science and Technology (UST), Daejeon, 34113 South Korea

## Abstract

Artificial olfactory sensors that recognize patterns transmitted by olfactory receptors are emerging as a technology for monitoring volatile organic compounds. Advances in statistical processing methods and data processing technology have made it possible to classify patterns in sensor arrays. Moreover, biomimetic olfactory recognition sensors in the form of pattern recognition have been developed. Deep learning and artificial intelligence technologies have enabled the classification of pattern data from more sensor arrays, and improved artificial olfactory sensor technology is being developed with the introduction of artificial neural networks. An example of an artificial olfactory sensor is the electronic nose. It is an array of various types of sensors, such as metal oxides, electrochemical sensors, surface acoustic waves, quartz crystal microbalances, organic dyes, colorimetric sensors, conductive polymers, and mass spectrometers. It can be tailored depending on the operating environment and the performance requirements of the artificial olfactory sensor. This review compiles artificial olfactory sensor technology based on olfactory mechanisms. We introduce the mechanisms of artificial olfactory sensors and examples used in food quality and stability assessment, environmental monitoring, and diagnostics. Although current artificial olfactory sensor technology has several limitations and there is limited commercialization owing to reliability and standardization issues, there is considerable potential for developing this technology. Artificial olfactory sensors are expected to be widely used in advanced pattern recognition and learning technologies, along with advanced sensor technology in the future.

## Background

Biomimetics is an interdisciplinary field in which engineering, chemistry, and biology principles are applied to the synthesis of materials, synthetic systems, or machines that have functions mimicking biological processes [1]. Humans have continuously attempted to design technologies that resemble nature. Weapons, such as spears and knives, used by primitive people were inspired by predators with sharp claws and teeth. Ancient Greeks saw sharp backbones of fish and made saws. They also used spider webs to stop bleeding when they saw how spiders used their webs to capture food. Why do humans study technology that resembles that of nature? It is because the excellent characteristics of these creatures enabled their survival. Recently, research on the application of biomimetic technologies has become increasingly active in various fields, including self-healing ability, environmental exposure resistance, hydrophobicity, self-assembly, and solar energy utilization [2–5].

Various human senses are digitized through sensors, and biomimetic technology with sensory recognition mechanisms has permeated into our daily life in various ways. Examples include image sensors that replace eyes, speakers that replace hearing, and pressure sensors that replace touch. In many instances, these sensors are much more sensitive than our sense organs, enabling visualization of infrared and ultraviolet radiation and making ultrasonic sound discernible [6–8]. Furthermore, these sensors also provide new senses such as a sense of location and orientation enabled by the global positioning system (GPS) and gyroscopes, respectively [9, 10]. However, there is surprisingly slow progress in the digitized detection of chemicals such as via smell and taste. This can be ascribed to the complexity of recognizing olfactory information and various technical limitations. However, interest in research on olfactory recognition mechanisms has been steadily increasing. Although our understanding of the mechanisms underpinning the sense of smell remains elusive, our limited knowledge is continuously being applied in various fields, such as the food, beauty, and health industries [11–13].

Smell is an important factor for survival. If there is limited vision and hearing, animals rely on their sense of smell to make situational judgments. Smell enables the detection of food and toxic fumes, including smoke from fires, at very long distances. Although the human sense of smell has deteriorated compared to that of other animals, it remains the most sensitive of the five senses. Visual senses can distinguish colors with three types of receptors, but olfactory senses can distinguish 10,000 kinds of smells with approximately 400 types of receptors [14, 15]. Olfactory receptors are proteins that bind to odor molecules in the nose, allowing the sensing of odors [16, 17]. A person with a well-developed sense of smell can detect odors diluted as low as 0.01 ppb (part per billion). It can detect one in 100 trillion air molecules [18, 19]. This is superior to state-of-the-art gas sensor technology, and artificial olfactory model research that imitates the olfactory mechanism through pattern recognition is emerging as a promising research field. Furthermore, owing to the importance of the sense of smell, the development of technology to reproduce it is also continuing. In the past, the sense of smell has been classified as a field that is difficult to investigate with scientific rigor. This is because it is very difficult to effectively collect and accurately distinguish odors. In addition, there are more than tens of thousands of types of odor, and its recognition is diverse. Therefore, research on the olfactory system has been slow to progress compared to that of the other senses.

Artificial olfactory systems have fascinated scientists for approximately 40 years, especially with a salient paper published by Persaud and Dodd in 1982 [20]. They showed that different odors can be distinguished using four chemical sensors with overlapping selection patterns. The signal combination pattern that appears in the ensemble of each receptor is key to the classification, identification, and recognition of odors [21]. Since the 1980s, almost all olfactory sensor technologies have consisted of sensor arrays with specific functions for classifying odors. An electronic nose (e-nose) model that imitates the human olfactory recognition system using an array of electronic sensors has been introduced and is emerging as a representative example of an artificial olfactory system [22].

**Fig. 1:**
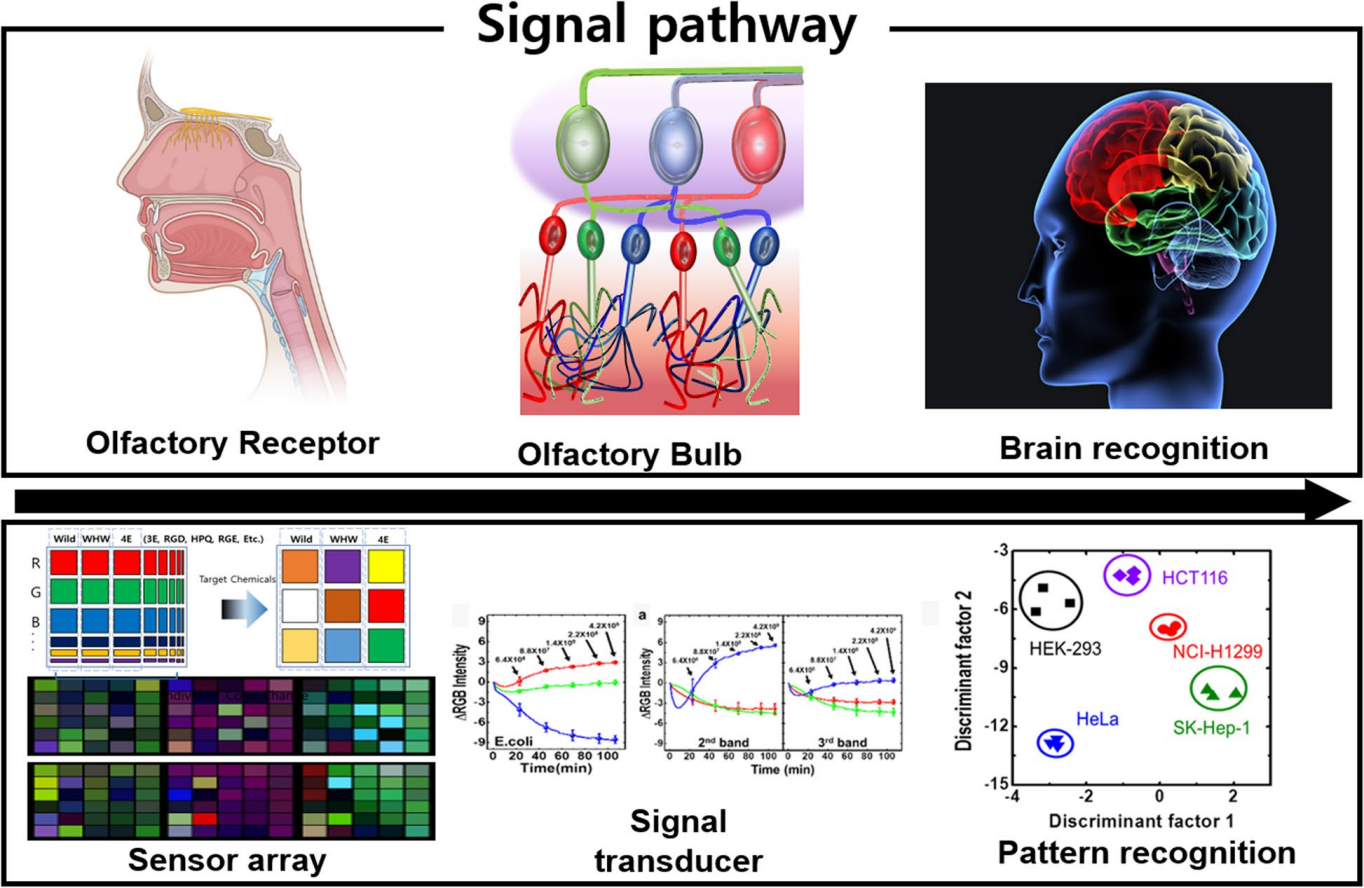
Schematic of biomimetic olfactory sensor based on olfactory recognition system. Development of artificial olfactory sensor systems through pattern recognition of sensor arrays, focusing on the mechanism by which humans detect and recognize odors

Thus far, research has mainly focused on nanosensor-based artificial receptor technology that can detect chemicals. Olfactory receptors are only one aspect of the rich architecture responsible for the sense of smell. However, other important functions of the olfactory epithelia, such as large numbers of olfactory receptor neurons, hierarchical organization, odor patterns in the olfactory bulbs (OBs), and the diffusion of odor molecules along the olfactory area, have been much less considered. To improve the development of artificial olfactory technology, it is necessary to understand the olfactory recognition mechanism and utilize engineering technology that can imitate it. Figure 1 presents a scheme of an artificial olfactory sensor model based on the human olfactory recognition system. This review introduces the biological olfactory recognition mechanism, the data processing technology of signal patterns that can imitate it, and discuss the research trends in artificial olfactory sensor system technology.

### Mechanism of olfactory recognition through pattern classification

#### Olfactory recognition process through pattern recognition of olfactory receptors

**Fig. 2:**
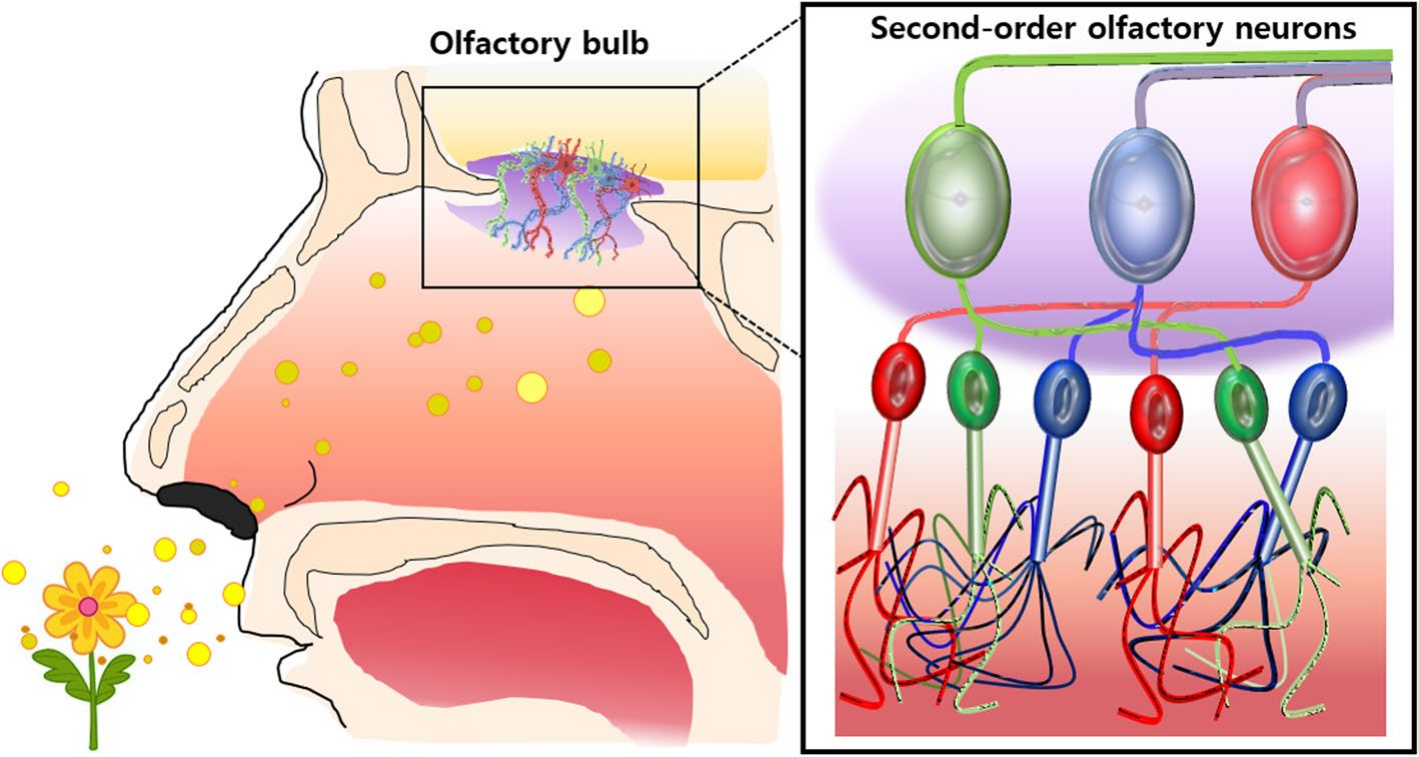
Schematic diagram of the olfactory system. In the process of inhaling air, volatile molecules reach the inside of the nose. The olfactory epithelium in the nasal cavity interacts with these odor molecules. The axons of the olfactory sensory neurons are projected onto the OB to be septaped with the dendrite of the secondary neuron, which is projected onto the olfactory cortex. The determination of smell is determined by the pattern formed by a combination of different receptors that recognize the specific molecular characteristics of each odor molecule

Menini et al. described the interaction of volatile molecules with various molecular structures in the vertebrate olfactory system (Fig. 2) [23]. In the process of inhaling air, volatile molecules reach the inside of the nose. The olfactory epithelium in the nasal cavity interacts with these odor molecules. Olfactory sensory neurons that act as receptors transmit molecular binding processes to the brain via electrical signals. Therefore, the essence of odor perception involves the transformation of the chemical interaction of olfactory receptors with volatile molecules into electrical signals that carry information about the external world to the brain [23, 24]. The information sent to the secondary neuron of OB is projected into the olfactory cortex and other brain regions. Information about odors is encoded in pattern form in OB. In other words, the determination of smell is determined by the pattern formed by a combination of different receptors that recognize the specific molecular characteristics of each odor molecule.

The sensitivity and accuracy of the biological olfactory system are excellent. Tens of thousands of low-molecular-weight organic compounds can be detected and differentiated. The olfactory system recreates memories of various organic polymers, including alcohols, esters, carboxylic acids, ketones, sulfides, nitriles, thiols, immigration, halides, and ethers [25] With the rise of cognitive engineering over the last 20 years, the sophisticated mechanism of smell has attracted attention.

From flies to mammals, higher eukaryotes developed a sense of smell in a common manner. Odor discrimination arises from the interaction between odor determinants and receptor residues [26]. The neural mechanism of odor discrimination begins with differential interactions between different types of receptors and odor molecules, similar to the interactions between antigens and antibodies in the immune system or between neurotransmitters and receptors in the nervous system [27, 28]. Similar to antigens, odor molecules can be called odogens and play the same role as epitopes. When a receptor binds to an odor molecule, it converts the chemical energy of the recognition event into a nerve signal (via a change in membrane potential). Signals transmitted by the olfactory receptors are transmitted through the OB. The OB is a structure that processes information about odors and is a key part of the nervous system responsible for the sense of smell. It serves as a pathway that transmits odor inputs to other locations in the brain. Smell is perceived through a combination of several olfactory receptors [29]. The brain recognizes each odor molecule as a unique combination code. Even a slight change in the structure of the odor molecule is recognized as a different odor owing to a different combination code. The signaling process of the OB is the most important factor, but its exact functional role remains uncertain [30, 31].

In 2015, Gschwend et al. reported that neuronal pattern separation in the OB improves odor discrimination [32]. They showed that a similar pattern of odor molecules induces a highly correlated input pattern in the OB. This indicates that pattern separation in the olfactory system acts as a driving force for sensory discrimination and learning. Wang et al. developed an artificial neural network (ANN) based on the mechanism by which the olfactory system classifies odors [25]. They reproduced the olfactory system of a fly using machine learning (ML). ANNs, which are capable of performing complex tasks, provide a novel approach for modeling neural circuits [33, 34]. Neural activity patterns from the higher visual areas of the brains of monkeys that are viewing natural images resemble those of neural networks trained to classify many visual images [34]. Many other studies have applied the mechanisms of natural olfactory detection by incorporating the pattern recognition process into engineering olfactory sensor technology.

#### Data processing of artificial olfactory sensor system

To imitate the olfactory recognition mechanism, sensor array technology that can transmit patterned signals for volatile organic compounds (VOCs) and data processing technology using artificial intelligence (AI) technology are essential. The multichannel sensor array functions as an olfactory receptor tissue and as the number of signal receptors increases, advanced signal classification through various patterns becomes possible. Therefore, it is necessary to introduce a systematic data analysis algorithm for the processing of high-dimensional pattern data.

Typically, two algorithms are used to process data obtained from artificial olfactory sensors, namely statistical and intelligent model analyses. Statistical pattern analysis methods include linear analysis, principal component analysis (PCA), linear discriminant analysis (LDA), and support vector machine (SVM). Intelligent model analysis includes ANNs, multilayer perceptron (MLP) and k-nearest networks (kNNs).

A multivariate analysis of the sensor arrays is required for specific chemical gases with mixed compositions. This analysis method is often used to visually distinguish between groups of the same sample in a PCA plot [35]. A PCA plot is a two-dimensional representation of the data, including the maximum variance of the data. An intelligent olfactory sensor can be implemented through advanced data processing techniques that use AI and ML using preprocessed data [11].

Zeng et al. performed PCA on the pattern data of a multiparameter virtual sensor array (VSA) for the discrimination of six VOCs. They found that two principal components could account for more than 97% of the accuracy [36]. PCA reduces redundancy within the sensor sensitivities for each class and projects the sensitivity data orthogonally to several unrelated dimensions to identify the maximum variable component that can be used to classify groups of odorants. The six VOCs datasets projected onto the main plane identified distinct groups of each VOC. Lu et al. performed PCA using a Quartz crystal microbalance (QCM) sensor for 15 VOCs to observe the discrimination of VOCs. Two PCs accounted for 95% of the variance in the sensitivity data, and the predictions of the two PCs showed a clear differentiation of VOCs [37]. PCA is mainly used to reduce high-dimensional data to low-dimensional data. An orthogonal transformation is used to transform highly correlated samples in high-dimensional space into low-dimensional space (PCs) that are not linearly correlated. When data are mapped to one axis, the data are linearly transformed into a new coordinate system such that the axis with the largest variance is placed as the first PC and the axis with the second largest variance as the second PC. This decomposition of sample differences into the components that best represent them provides several benefits for data analysis.

A supervised learning method is mainly used to establish a functional relationship between the measurement space and classification element. Many ML methods have been developed over the past few decades, including partial least squares (PLS) regression, SVMs, ANNs, decision trees (DTs), and kNNs [38–42]. Among these, neural networks such as MLPs have been widely used [43]. ANNs are currently the most common applications of AI. Through these algorithms, it is possible to automatically detect patterns in data, predict or classify future data using undiscovered patterns, and derive new knowledge by collecting or extracting information from suitable data.

Statistical and artificial neural network-based nonlinear pattern recognition models based on LDA and orthogonal discriminant analysis (ODA) have been applied to food contamination discrimination technology [44–48]. ANNs are statistical learning algorithms that are inspired by biological neural networks (particularly the brain in the central nervous system of animals) and are frequently used in ML and cognitive science. An ANN refers to an overall model in which artificial neurons (nodes) form a network through synaptic bonding. Through learning, the bonding strength of synapses change and develop problem-solving abilities [49].

ANNs rely on many inputs and are generally used to guess and approximate hidden functions. It is usually represented as an interconnection of neuronal systems that computes values from inputs and is adaptable, allowing ML tasks such as pattern recognition to be performed. Similar to other ML systems, learning from data-neural networks is used to solve a wide range of problems, such as computer vision and speech recognition, which are typically difficult to solve using rule-based programming [50].

An ANN is also a biomimetic technology and is used to classify and predict results based on the similarity and closeness among complex and nonlinear data [51]. Probabilistic neural networks (PNNs), radial basis function networks (RBFNs), back-propagation neural networks (BPNNs), and SVMs have been used to classify VOCs [52–55]. An ANN is typically composed of an input layer, hidden layer, and decision (output) layer. The input layer accepts data patterns to transmit data to the hidden layer nodes. The hidden layer node performs a learning process to determine the data. In general, the higher the number of nodes in the hidden layer, the better the classification of complex input data achieved through training [56]. In the ANN learning process, an optimal weighting process that connects the hidden and output layers is performed. The roles of step, momentum, quick propagation, delta bar delta, conjugate gradient, and Levenberg–Marquardt (LM) are used for ANN training [57]. The LM method does not rely on the initial weights for network convergence and uses second-order gradients to allow the network to converge faster. Thus, the LM method is more stable, efficient, and faster in learning than backpropagation-based learning [58] More information related to material classification and identification using pattern recognition technology can be found in Karakaya et al. [59], Wasilewski et al. [60], .Tonacci et al. [61], and Sanaefar et al. [62].

### Artificial olfactory sensor model based on natural olfactory recognition system

Because the artificial olfactory sensor system is capable of quantitative and qualitative analysis of chemical gases, it can be used in industrial fields that require regular monitoring for safety [63, 64]. The e-nose system is based on a chemical sensor unit that converts chemical information into a digital signal, forming an array capable of providing a multi-dimensional response when it comes into contact with specific VOCs. To relate specific recognition events to specific VOCs, multi-modal sensor array technology and multi-dimensional pattern recognition data processing technology are required.

#### Sensor arrays for e-nose system

**Fig. 3:**
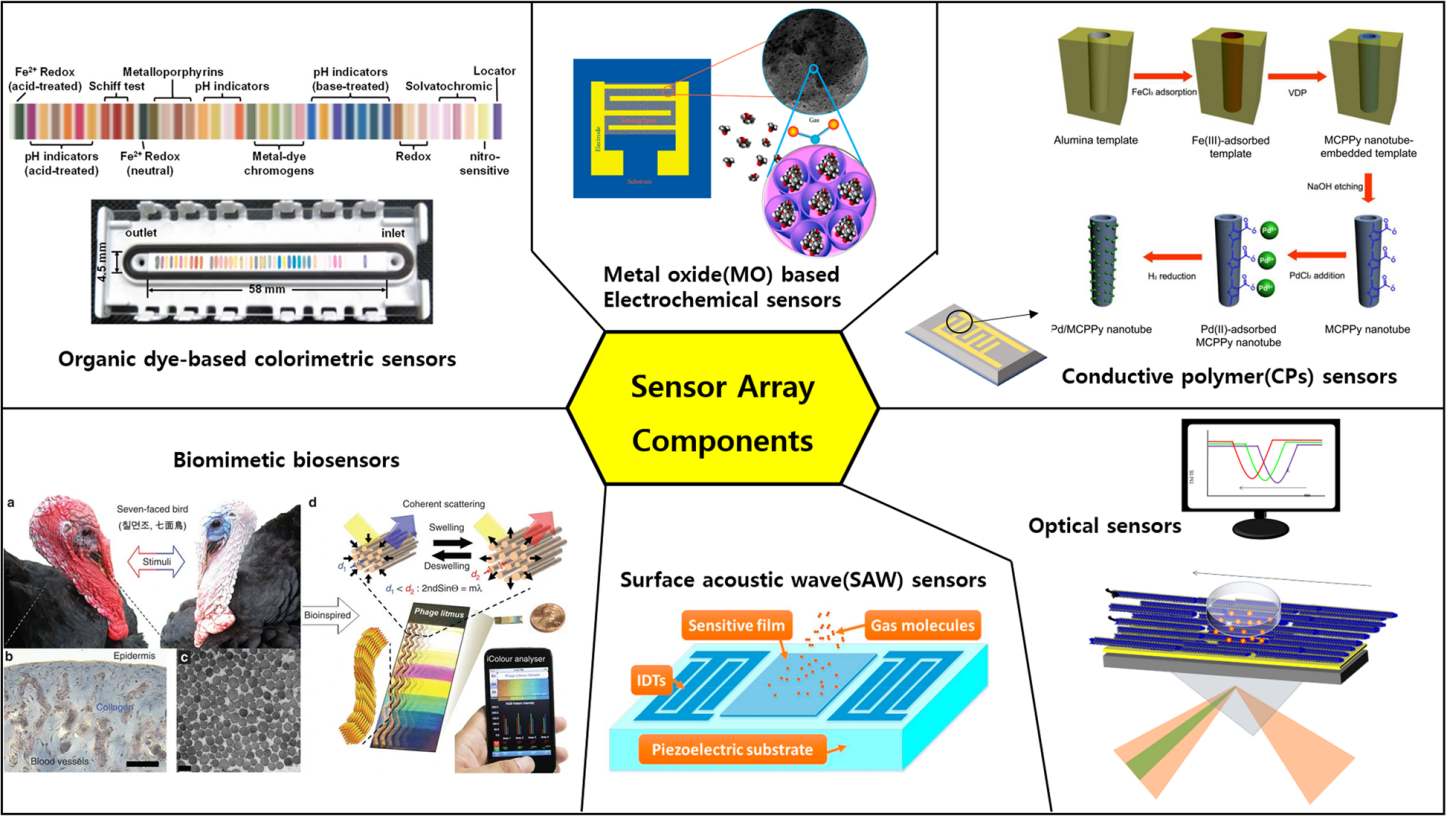
Various sensor technologies that can be used as units for multi-array sensors [65–70]

In artificial olfactory systems, various nanosensor technologies are used as units that make up the sensor array. Any sensor platform for which its array can form its own pattern is available. Figure 3 introduces various sensor technologies that can be used as units of arrays that can form patterns. Available sensors include metal oxide (MO)-based electrochemical sensors, surface acoustic waves (SAW), conductive polymers (CPs), organic dye-based colorimetric sensors, biomimetic biosensors, optical sensors, and mass spectrometry (MS) [62]. Table 1 provides information on the sensor platforms that can be used as sensor array units. Further information on the sensor technology being used as an e-nose system can be found in Kim et al., Zheng et al., Jha et al., Nazemi et al., Feng et al., and Hangxun et al. [13, 65, 86–89].

**Table 1:** Characteristics of commonly used sensor units

Sensor type	Strengths	Weaknesses
Metal oxide (MO)-electrochemical sensors [71–73]	High sensitivity, target diversity, short response time, easy to dissociate, convenient replacement	High energy required, inaccurate readings (sensor drift), controlled environment, controlled setting (vacuum), streaky fabrication
Surface acoustic waves (SAW) [74, 75]	High sensitivity, target diversity, short response time, diverse range of coatings, concise configuration	High cost, high energy required, complex circuitry, commercialization, controlled temperature, reproducibility
Conductive polymer (CP) [66, 67]	High sensitivity, short response times, low cost, room temperature operation, diverse range of coatings	Low durability (weak), inaccurate readings (sensor drift), complex synthetic process
Organic dye-based colorimetric sensors [76, 77]	Excellent intuition, small, no external power required, portable, convenient	Low sensitivity, complex manufacturing process
Biomimetic biosensors [78–82]	Excellent intuition, small, no external power required, portable, convenient, high sensitivity, high selectivity, wide compatibility, eco-friendly	Lack of standardization, limited mass production
Optical sensors [83–85]	Very high sensitivity, low energy consumption, individual response (compounds mixture analysis), quick response.	High cost, complex construction, difficult to make portable system
Mass spectroscopy (MS) [76, 77]	Short response time, high sensitivity and stability, enables qualitative and quantitative analysis, universal detector	High cost, complex construction (spectrometer), response time, difficulty of field analysis

#### Applications of artificial olfactory systems

From the aforementioned discussion, it is possible to build an artificial olfactory system that imitates the natural olfactory structure by combining gas sensing and data processing technologies. Artificial olfactory sensor systems have been actively researched and developed. Table 2 introduces the current status of research on such e-noses, which are widely applied in various fields.

**Table 2:** Applications of gas discrimination using artificial olfactory sensors in various fields

Applications	Contents	Sensor unit	Data process	Reference
Food science	Detection of beef freshness	Cyranose-320™: MO-based 8 sensor arrays	Principal component analysis (PCA), linear discriminant analysis (LDA), quadratic discriminant analysis (QDA)	[45]
	Quality assessment of modified-atmosphere packaged poultry meat	MO-based 24 sensor array	PCA, partial least squares (PLS), artificial neural network (ANN)	[90]
	Contaminations in tomatoes	EOS835 (Sacmilmola scarl, Italy): MO-based 6 sensor array	PCA, k-nearest network (kNN)	[91]
	Descriptive sensory analysis of aged cheddar cheese	Gas chromatography (GC)-based sensor array	PCA	[92]
	Portable electronic nose device to determine the freshness of Moroccan sardines	MO-based 6 sensor arrays	PCA, support vector machine (SVM)	[93]
	Monitoring of growth of spoilage bacteria in milk	10 MO semiconductor field effect transistor (MOSFET) sensors	PLS	[94]
	Freshness monitoring of peach	Structural colorimetric sensors array	Hierarchical classification analysis (HCA)	[95]
	Banana ripening	Functional bacteriophage-based colorimetric sensor array	HCA, PCA	[80]
Environmental monitoring	Automobile exhaust	MO-based sensor array	Back-propagation neural network (BPNN)	[96]
	Physical discrimination of amine vapor mixtures	Polymer-based thin film transistor (TFT) sensor array	Extracting values from data curves	[97]
	BTX (Benzene, toluene, xylene) vapors in Air	SAW sensors	PCA, probabilistic neural networks (PNN)	[98]
	NOx urban pollution monitoring	MO-based sensor array	ANN	[99]
	Aromatic hazardous chemicals	Functional phage-based colorimetric sensor array	ANN, HCA	[78]
	Hydrogen sulfide and nitrous oxide detection	MO sensor array	PCA, discriminant factorial analysis (DFA)	[100]
	Highly polluted river	CP sensor array	PCA	[101]
	Antibiotics pollution in water	Biomimetic colorimetric sensor	PCA, LDA	[102]
	Endocrine-disrupting chemicals detection	Biomimetic colorimetric sensor	PCA, LDA	[103]
	Pharmaceutical chemicals discrimination	Biomimetic colorimetric sensor	HCA	[104]
Diagnostics	Breath diagnosis for lung cancer (LC) and lung disease	Quartz crystal microbalance (QCM) sensor array	Partial least squares discriminant analysis (PLS-DA)	[105]
	LC, gastric cancer, asthma, and chronic obstructive pulmonary disease	Silicon nanowire sensors	ANN	[106]
	Exhaled breath diagnosis for LC	Graphene oxide sensor array	ANN	[107]
	Chronic liver disease	Bionote (Commercial e-nose devices) [108]	PLS-DA	[109]
	Chronic kidney disease	MO-based 11 sensor arrays	SVM	[110]
	LC	Functional phage-based colorimetric sensor array	ANN	[111]
	Ventilator associated pneumonia	MO sensors	Logistic regression analysis	[112]

#### Artificial olfactory system applications in food science

There is a growing demand for fast and accurate quality testing of food. Currently, daily evaluation of food is conducted through visual inspection and subjective evaluation. Artificial olfactory sensor systems offer innovations in the non-destructive quality assessment of agricultural products and foods [113]. Taste receptors produce a sense of smell in response to these types of foods based on volatile compounds. Flavors are mostly derived from VOCs [114]. Electronic nose technology, which can analyze the composition of VOCs and identify specific types of aromatic chemicals, enables an objective assessment of food conditions.

Panigrahi et al. verified the freshness of beef using a commercially available e-nose device (Cyranose-320). Dimensions were reduced by performing PCA on the signal transmitted by the sensor array and 100% discrimination between undamaged samples and those damaged by microorganisms [45]. Rajamaki et al. conducted an experiment using an e-nose device to detect spoilage signals in packaged poultry meat at an early stage. Gas chromatography (GC) confirmed that the composition of hydrogen sulfide and dimethyl sulfide differed depending on the freshness of meat. The signal output from the sensor array was processed using PCA, PLS, and ANN, and similar classification results were obtained among the method [90]. Concina et al. used an e-nose sensor to determine microbial contamination of tomatoes. The composition of the VOCs mixture consisting of 3-methyl-furan, dimethyl sulfide, acetone, ethanol, and 3-methyl-1-butanol was classified using PCA and kNN [91]. Drake et al. performed a descriptive sensory analysis of aged cheddar cheese using an e-nose equipped with an MS(Mass-spectrometer) detector. PCA was found to provide a simple chemical basis for distinguishing cheddar cheese flavors [92]. Barbri et al. proposed an e-nose model in the form of a MO-based sensor array to develop a portable device that can determine the freshness of Moroccan sardines [93]. PCA and SVMs results showed that the system was able to assess the freshness of sardines stored at 4 °C. Haugen et al. performed a study on the detection and monitoring of the growth of spoilage bacteria in milk [94]. The composition of VOCs in milk generated by three putrefactive bacteria (*Serratia marcescens*, *Serratia proteamacufans*, and *Pseudomonas putida*) was elucidated using a 10 MO semiconductor field effect transistor (MOSFET) sensor array. They compared the gas profile measured by the e-nose with combined GC-MS analysis results and confirmed that the prediction was possible with an error of less than 5%. Lee et al. produced an amino acid-based colorimetric sensor array that could discriminate various VOCs [95]. They created a sensor array that could discriminate between six types of VOCs (Y-hexanolactone, 2-isopropyl-4-methylthiazole, ethanol, acetone, ethyl acetate, and acetaldehyde) using a series comprising tryptophan and histidine residues. They could monitor the freshness of peaches using this sensor. Kim et al. produced a sensor array using a colorimetric sensor based on functional bacteriophages and developed an artificial olfactory sensor model that could identify the ripening state of bananas with 95% accuracy [80]. Artificial olfactory sensors are widely applied in fields that can quickly detect the condition of foods.

#### Artificial olfactory system applications in environmental monitoring

Electronic nose models are widely used in the field of environmental monitoring. Although humans can react to hazardous situations by recognizing odors, the natural olfactory system can easily tire [115, 116]. It is difficult to continuously measure bad odors in the field, making e-nose technology essential. Environmental monitoring technology requires 1) the ability to standardize VOCs mixtures, 2) signal transmission for non-specific chemical gas exposure, and 3) high sensitivity. Commercial environmental monitoring sensors are currently in limited use because they are lacking in many areas that need to be improved upon, such as high durability, repeatability, standardization, and detection limits, to enable operation in poor environmental conditions [117–119]. Because environmental regulations have been strengthened and issues raised in recent decades, the demand for environmental monitoring technology is increasing rapidly.

Wang et al. developed a powerful vehicular e-nose system for detecting automobile exhaust gases such as carbon monoxide and hydrocarbons [96]. An ANN-based gas pattern recognition method was used to improve the selectivity of the gas sensors and accurately discriminate the gas components. The classification of emitted gases was based on a momentum and adaptive learning rate BPNN, whose weights and biases were trained in advance and programmed in a microcontroller unit (MCU). The experimental results demonstrate that the system can not only effectively detect the individual components from their mixtures, but also monitor the risk level of each gas with sufficient accuracy. Liao et al. conducted a study on the physical identification of amine vapor mixtures using polythiophene gas sensor arrays. By varying the side chain of the polythiophene molecule and adjusting the thickness of the polythiophene films, size discrimination of amine vapors could be accomplished using small arrays of polythiophene transistors [97]. Matatagui et al. reported a SAW sensor-based e-nose device array that can identify and monitor benzene, toluene, and xylene in air [98]. They reduced the dimensions of the pattern data using PCA. PNN learning was performed based on the classification results of PC1, PC2, and PC3, and was repeated until all vectors were verified, showing 100% accurate classification. Lee et al. developed an e-nose device that can detect harmful aromatic chemicals based on a neural network method [78]. Their neural pattern separation mimics the mammalian olfactory system with detection possibilities close to the K-9 level. A highly distinguishable detection rate at the atomic level resulted in a high selectivity rate of 97.5%.

#### Artificial olfactory system applications in diagnostics

Diagnosis through exhaled breath analysis, inspired by the traditional method of checking the disease state through the smell of the patient’s breath, is an emerging field of research as e-nose technology advances. Junqueira et al. reported on a cancer diagnosis study using trained dogs [120]. Investigations of exhaled breath analysis mainly concern the diagnosis of respiratory diseases such as lung cancer (LC), chronic obstructive pulmonary disease, and asthma [121, 122]. Because it is well known that various diseases affect metabolic mechanisms, monitoring VOCs composition changes in the exalted breath is expected to become widely used to diagnose various diseases [13, 123].

A sample-based diagnostic method, such as a patient’s blood or tissue, 1) is a cumbersome process, 2) requires a skilled operator due to a difficult protocol, 3) can be performed only in a facility such as a hospital, and 4) has long analysis times. However, disease diagnosis using breath analysis is user-friendly, non-invasive, and real-time analysis is possible.

LC is the foremost target for breath diagnoses. Studies on the variation of VOCs occurring in the process of cancer cell culture were conducted by Moon et al. and Thriumani et al. [79, 124]. Their findings indicate that the specific VOCs released from cancer cells can act as odor signatures and potentially be used for the non-invasive screening of LC using gas array sensor devices. Lee et al. published a study on the classification of exhaled breath from 31 patients with LC and 31 healthy subjects using an e-nose based on a phage colorimetric sensor [111]. With the help of deep learning and neural pattern separation, the e-nose achieved a diagnostic success rate of over 75% and a classification success rate of over 86% for LC based on raw human breath data.

Hanson et al. conducted a study to predict the clinical pneumonia score using an e-nose. They aimed to determine whether exhaled breath analysis correlated with the clinical pneumonia score [125]. Exhaled gas was sampled from the expiratory limb of the ventilator in mechanically ventilated surgical intensive-care patients and assessed using an e-nose. The components of the clinical pneumonia score were concurrently recorded. The e-nose score showed a correlation with the clinical pneumonia score. Amal et al. collected 968 breath samples from 484 patients (including 99 with gastric cancer) and analyzed them through GC-MS and an e-nose array [126]. According to the GC-MS results, patients with cancer and those at high risk had distinctive breath-print compositions. Eight significant VOCs (*p* < 0.017) were detected in the exhaled breath. The nanoarray analysis made it possible to discriminate between patients with gastric cancer and the control group (OLGIM 0–IV) with 73% sensitivity, 98% specificity, and 92% accuracy.

Hakim et al. conducted a head-and-neck cancer (HNC) diagnostic study using an e-nose device [127]. HNC is the eighth most common malignancy in the world. It is often diagnosed late because of the lack of screening methods, and complete remission is achieved in < 50% of patients. HNC patients often develop a second primary tumor that can affect the entire aerodigestive tract, necessitating lifelong follow-up. The e-nose could clearly distinguish between (i) HNC patients and healthy controls, (ii) LC patients and healthy controls, and (iii) HNC and LC patients.

Xu et al. studied the feasibility of a nanomaterial-based breath test to identify gastric cancer in patients with gastric complaints [128]. The models were insensitive to the confounding factors tested. Chemical analysis revealed that five VOCs (2-propenenitrile, 2-butoxy-ethanol, furfural, 6-methyl-5-hepten-2-one, and isoprene) were significantly elevated in patients with gastric cancer and/or peptic ulcers compared to those with less severe gastric conditions. The concentrations in both room air and breath samples were in the single ppb range, except in the case of isoprene. Upper digestive endoscopy with biopsy and histopathological evaluation of biopsy material is the standard method for diagnosing gastric cancer. However, this procedure may not be widely available for screening in the developing world, whereas endoscopy is frequently used without major clinical gain in developed countries. There is a high demand for a simple and non-invasive test for screening individuals at increased risk that should undergo endoscopic examination.

Amal et al. developed a diagnostic breath test that could distinguish between patients with malignant ovarian tumors and those who were tumor-free [129]. The test used a nanoarray of sensors to measure VOCs; it showed good sensitivity (low false negatives) and 100% specificity (no false positives). This may lead to an inexpensive and disposable alternative for the early diagnosis of ovarian cancer. Because ovarian cancer is usually not diagnosed until it reaches an advanced stage, its mortality rate is very high. The current diagnostic tests are expensive and cumbersome, making widespread screening impractical, highlighting the need for a rapid analysis such as e-nose technology.

## Conclusions

Herein, we reviewed artificial olfactory sensor technology based on natural olfactory mechanisms. Configuration technology for the engineering of artificial olfactory sensors was described. Compared to other sensory systems, the olfactory mechanism is the most undetermined as it is the most complex. Advances in computer and data processing technologies have led to the development of biomimetic olfactory sensing technology in the form of pattern recognition. ANNs based on biological recognition mechanisms have led to the development of artificial olfactory sensors. This statistical method enables the classification of a larger number of sensor arrays and the analysis of more complex data. Electronic nose technology is a typical example of artificial olfactory sensors. Sensor array units that act as olfactory receptors can be applied to various nanosensors. MO-based electrochemical sensors, SAW, QMC, CPs, organic dye-based colorimetric sensors, biosensors, optical sensors, and MS devices are utilized as units that make up the sensor array. Each sensor platform has its advantages and disadvantages. However, an array can be tailored to meet the operating environment and performance requirements of the artificial olfactory sensor. The artificial olfactory sensor system can be used to analyze the chemical composition and quantitative and qualitative levels of trace VOCs in a wide range of fields such as food quality and safety evaluation, environmental monitoring, and diagnosis. In particular, artificial olfactory sensor technologies related to diagnosis have recently been highlighted. Utilizing exhaled-breath analysis can lead to the development of efficient and reliable real-time inspection techniques. It has a high potential as a screening tool for early non-invasive diagnosis. However, the current artificial olfactory sensor technology has several limitations and there is limited commercialization owing to reliability and standardization issues. However, prospects for the development of this technology are positive. Artificial olfactory sensors are expected to be widely used in advanced pattern recognition and learning technologies, along with advanced sensor technology in the future. Through the integration of Internet of Things and artificial olfactory sensors technologies, it is expected that artificial olfactory sensors that can be used in mobile wearable devices can permeate our daily lives.

## Data Availability

Not applicable.

## Abbreviations

AI: Artificial intelligence
ANN: Artificial neural network
BPNN: Back-propagation neural network
CP: Conductive polymers
GC: Gas chromatography
HCA: Hierarchical classification analysis
HNC: Head-and-neck cancer
kNN: k-Nearest network
LC: Lung cancer
LDA: Linear discriminant analysis
ML: Machine learning
MLP: Mltilayer perceptron
MO: Metal oxide
MS: Mass spectrometry
OB: Olfactory bulbs
ODA: Orthogonal discriminant analysis
PCA: Principal component analysis
PLS: Partial least squares
PNN: Probabilistic neural network
QMC: Quartz crystal microbalance
RBFN: Radial basis function network
SAW: Surface acoustic wave
SVM: Support vector machine
VOC: Volatile organic compound
VSA: Virtual sensor array
